# CAR-T manufactured from frozen PBMC yield efficient function with prolonged *in vitro* production

**DOI:** 10.3389/fimmu.2022.1007042

**Published:** 2022-09-26

**Authors:** Julieta Abraham-Miranda, Meghan Menges, Reginald Atkins, Mike Mattie, Justyna Kanska, Joel Turner, Melanie J. Hidalgo-Vargas, Frederick L. Locke

**Affiliations:** ^1^ Department of Clinical Science, H. Lee Moffitt Cancer Center and Research Institute, Tampa, FL, United States; ^2^ Kite Pharma, A Gilead Company, Santa Monica, CA, United States; ^3^ Department of Blood and Marrow Transplant and Cellular Immunotherapy, H. Lee Moffitt Cancer Center and Research Institute, Tampa, FL, United States

**Keywords:** chimeric antigen receptor T-cell, CAR-T cell, PBMC cryopreservation, CAR-T cell *in vitro* expansion, CAR-T cell cytokines production

## Abstract

Chimeric antigen receptor (CAR)-T cells are engineered to identify and eliminate cells expressing a target antigen. Current manufacturing protocols vary between commercial CAR-T cell products warranting an assessment of these methods to determine which approach optimally balances successful manufacturing capacity and product efficacy. One difference between commercial product manufacturing methods is whether T cell engineering begins with fresh (unfrozen) patient cells or cells that have been cryopreserved prior to manufacture. Starting with frozen PBMC material allows for greater manufacturing flexibility, and the possibility of collecting and storing blood from patients prior to multiple lines of therapy. We prospectively analyzed if second generation anti-CD19 CAR-T cells with either CD28 or 4-1BB co-stimulatory domains have different phenotype or function when prepared side-by-side using fresh or cryopreserved PBMCs. We found that cryopreserved PBMC starting material is associated with slower CAR-T cell expansion during manufacture but does not affect phenotype. We also demonstrate that CAR-T cell activation, cytokine production and *in vitro* anti-tumor cytotoxicity were not different when CAR-T cells were manufactured from fresh or cryopreserved PBMC. As CAR-T cell therapy expands globally, the need for greater flexibility around the timing of manufacture will continue to grow. This study helps support the concept that cryopreservation of PBMCs could be the solution to these issues without compromising the quality of the final CAR-T product.

## Introduction

Chimeric antigen receptors (CARs) are engineered synthetic receptors that redirect leukocytes, usually T cells, to identify and eliminate cells expressing a specific target antigen (1, 2). Anti-CD19 CAR-T cell therapy has produced effective and durable clinical responses in patients with refractory large B-cell lymphoma (3), which resulted in its approval by the US Food and Drug Administration (FDA) in 2017 (4). Currently, there are four FDA-approved CAR-T cell products targeting CD19 to treat B cells malignancies: axicabtagene ciloleucel, tisagenlecleucel, lisocabtagene maraleucel, and brexucabtagene autoleucel (5), and two anti-BCMA CAR-T cell products to treat refractory Multiple Myeloma: idecabtagene vicleucel (6) and ciltacabtagene autoleucel (7).

Manufacturing these cellular immunotherapies is a complex process that typically utilizes viral gene editing approaches to reprogram T cells. As a result, these protocols are highly regulated, and must follow Good Manufacturing Practice (GMP) guidelines, to ensure the release of safe and potent autologous CAR-T cell products (8, 9). This process begins when a patient undergoes leukapheresis to isolate peripheral blood mononuclear cells (PBMCs) from red blood cells and platelets. Isolated PBMC are then either shipped fresh or after cryopreservation to a manufacturing facility. When shipped fresh, manufacturing may begin immediately, or samples can be cryopreserved to allow for later manufacture if the patient needs a second infusion or the first attempt to manufacture the initial product fails. While protocols differ across products, manufacturing invariably involves T cell activation, transduction with a viral vector encoding the CAR, and *in vitro* expansion to reach target dose (10–12). After manufacture, all commercial CAR-T cells are cryopreserved and distributed to the clinical center where the patient receives their CAR-T cell infusion (10, 11). Cryopreservation of the CAR-T infusion product allows for completion of quality control testing and flexibility in shipping and treatment timing (9).

There is heterogeneity across products as well as differing practices for the same commercial product regarding whether the manufacture of CAR-T begins with a fresh or a cryopreserved PBMC product, which could impact CAR-T cell function and fitness. Manufacturing from frozen PBMC allows for greater manufacturing flexibility, maximization of required clean room facilities, and the possibility of collecting and storing blood from patients prior to frontline therapies with the purpose of having healthier T cells (8, 13). Alternatively, freezing the T cells prior to manufacture could decrease their ability to proliferate or secrete cytokines (14, 15).

Here we performed a prospective study to determine if second generation CD19-targeted human CAR-T cells with either CD28 (h1928z) or 4-1BB (h19BBz) co-stimulation, have differing phenotype or function when prepared side-by-side using fresh or cryopreserved PBMCs from same healthy donors.

## Materials and methods

### Cell lines and human peripheral blood mononuclear cells

K562 (CD19+ and null), 3T3 (CD19+ and null), and OCI-LY3 were used as target cells. Cells were tested for Mycoplasma using the MycoAlert PLUS Mycoplasma Detection Kit (Lonza) and were negative. Healthy donor PBMCs were obtained from buffy coats (LifeSouth Community Blood Centers). T cells were enriched from PBMCs using the EasySep human T cell isolation kit according to manufacturer’s instructions (STEMCELL) and stimulated *in vitro* using anti-CD3/anti-CD28 activation beads (Life Technologies 11132D). DMEM medium containing 10% FBS, 100 U/mL penicillin, 100 μg/mL streptomycin, and 2 mM L-glutamine was used to culture K562 and 3T3 cells, RPMI1640 medium containing 10% FBS, 100 U/mL penicillin, 100 μg/mL streptomycin, 2 mM L-glutamine for OCI-LY3 cells, and RPMI1640 medium containing 10% human serum, 100 U/mL penicillin, 100 μg/mL streptomycin, 2 mM L-glutamine, and 100UI/ml IL-2 for human PBMCs and T cells. All media and supplements were purchased from ThermoFisher Scientific.

### PBMC and CAR-T cell cryopreservation

50-100x10^6^ PBMC or 2-6x10^6^ CAR-T cells were cryopreserved in human serum supplemented with 5% (v/v) DMSO. Cells were collected in cryovials (SPL Life Sciences, Cat. #43022) and stored at -80°C overnight using a freezing container (Mr. Frosty™) filled with isopropanol. The next day cryovials were transferred to liquid nitrogen and stored until analysis (no more than 6 months).

### CAR-T cell manufacture

Retroviral supernatants encoding CARs were generated by modifying retroviral vectors to include human CD19-specific constructs containing the FMC63 scFv with CD8α hinge and trans-membrane domain followed by one co-stimulatory domain (either CD28 or 4-1BB) and CD3z. Both constructs included a non-functional truncated human CD34 and were transfected into H29 cells. RD114 cells were then transduced with retroviral supernatants collected from transfected H29. PBMCs or T cells were transduced using retroviral supernatant harvested from transduced RD114 cells. Briefly, buffy coats from healthy donors were processed in Ficoll to collect PBMCs and T cells were enriched using Stemcell Technologies kit.

When using bulk PBMCs as starting material, two million cells per well were added to a 6-well plate in 2ml media supplemented with 50ng/ml OKT3 antibody and 300IU/ml recombinant human IL-2 and incubated for 24h. Remaining PBMCs were frozen for later use. When using isolated T cells as starting material, T cells were first enriched using EasySep human T cell isolation kit (STEMCELL), then activated using CD3/CD28 dynabeads. After 24h, cells were washed, counted, and one million cells per well were added to a six well retronectin-coated plate in 1ml media supplemented with 200IU/ml recombinant human IL-2. Then, 1ml of either h1928z or h19BBz viral supernatant was added to each well containing either activated-PBMCs or activated-T cells and centrifuged at 2000xg for 60 minutes and 32°C (spinoculation). After 24h, 1ml of viral supernatant containing IL-2 was added to each well and the spinoculation step was repeated. After 24h, additional media supplemented with IL-2 was added to each well and cells were expanded for 8-12 days with fresh media. Then, cells were collected and frozen (“fresh” condition). Cryopreserved PBMCs from the same donor were thawed and, on the same day without resting them, the above protocol was repeated (“cryo” condition). Fresh and cryopreserved PBMCs from the same healthy donor were transduced using the exact same batch of viral supernatant to avoid using different retroviral titers in paired samples.

Cells were counted on an automated cell counter (Nexcelom Cellometer Auto 1000) and viability was detected by trypan blue staining. CAR-T cell transduction efficiency was estimated by flow cytometry as detailed below.

For functional experiments the number of CAR positive cells were normalized to the lowest CAR expression by adding un-transduced (UT) cells reaching the same number of CAR positive and total T cells per group.

### CAR-T cell thaw and rest

Cryopreserved CAR-T cells were removed from liquid nitrogen, and rapidly thawed in a 37˚C water bath prior to being transferred to 10ml of pre-warmed complete media to remove excess of DMSO. Cells were centrifugated 5 min at 1500rmp, the cell pellet was collected and resuspended in 1ml of fresh pre-warmed complete media. Cells were counted and rested for 24h in pre-warmed media containing 100IU/ml IL-2 at a concentration of 1M CAR-T cells per ml.

### CAR-T cell activation assay

Cryopreserved CAR-T cells were thawed, washed, and rested for 24h in media containing 100IU/ml IL-2 as described before. Then, CAR-T cells were collected, washed with PBS and 1x10^5^ CAR-T cells were co-cultured with irradiated K562-hCD19 or K562-null target cells at 5:1 E/T ratio for 72h in complete media supplemented with 100IU/ml IL-2. Cells were collected and markers of activation or metabolic fitness were analyzed by flow cytometry.

### Flow cytometry

Cells were collected, washed twice with PBS, and resuspended in 100µl of a solution containing 1X Live/Dead Fixable yellow cell stain (Invitrogen, ThermoFisher Scientific) and 1µL of human Fc block (BD). Cells were incubated for 30 min at room temperature. Surface staining was performed for 30 min at 4°C with antibody mix in MACS buffer with 0.5% BSA (Miltenyi Biotec). CAR expression on transduced cells was detected using an anti-FMC63 antibody (Acrobiosystems). The following monoclonal antibodies against human were obtained from BD Biosciences: anti-CD3 (UCHT1), anti-CD8 (SK1), anti-CD45RO (UCHL1), anti-CCR7 (150503), anti-4-1BB (4B4-1), anti-CD107a (H4A3), and anti-pAKT (M89-61).

For mitochondrial staining, cells were collected, washed, and resuspended in 100µL of PBS containing MitoTracker Red (25nM, Invitrogen), MitoTracker Green (25nM, Invitrogen), and 1X Live/Dead Fixable Near IR cell stain (Invitrogen, ThermoFisher Scientific), or in 100 µL of a PBS containing ApoTracker Green (400nM, Biolegend) and 1X Live/Dead Near IR cell stain. Cells were incubated at room temperature for 20 minutes. Then, cells were washed, and surface staining was performed in 100µL MACS buffer containing anti-FMC63 and anti-CD3 antibodies for 20 minutes at room temperature.

For staining phosphorylated proteins, CAR-T cells were stimulated with OCI-LY3 cells and stained with LIVE/DEAD Blue dye for 20 min at 37°C. Then, cells were fixed with Lyse-Fix buffer (BD) for 10 min at 37°C, washed, and then permeabilized with Perm Buffer III (BD) for 30 min on ice. Next, cells were stained with anti-CD3, anti-FMC63 and anti-pAKT for 30 min at room temperature.

Samples were analyzed with LSR II or Symphony flow cytometers (BD Biosciences) and data were analyzed using FlowJo software.

### ELLA

CAR-T cells were collected, washed with PBS to remove IL-2, and 2.5x10^4^ CAR-T cells were co-cultured with 3T3-hCD19 or 3T3-Null target cells at 1:1 E/T ratio for 24h. Supernatants were collected and stored at -80°C until analysis. Cytokines were measured using an ELLA Assay kit (Multianalyte: IFN-γ, IL-2, TNF-α and GM-CSF) according to the manufacturer’s instructions.

### Real-time cell analysis

Cytolysis assays were performed on Agilent xCELLigence Real-Time Cell Analysis (RTCA) MP instrument (ACEA Biosciences) following the manufacturer’s instructions. Briefly, 1x10^4^ 3T3-hCD19 target cells were seeded per well on an E-Plate 96. After 24h, 1 x10^4^ CAR-T cells in fresh complete medium, without IL-2, were added to target cell containing wells. Impedance, correlating to viable target cell growth, was measured every 15 minutes for 48 hours.

### Statistics

Prism 9 software (GraphPad) was used to execute all the statistical analyses. A P value ≤ 0.05 was considered significant. The statistical test used for each experiment is described in each figure legend.

## Results

### The manufacturing doubling time of CAR-T cells is longer when starting from frozen PBMC

**Figure 1:**
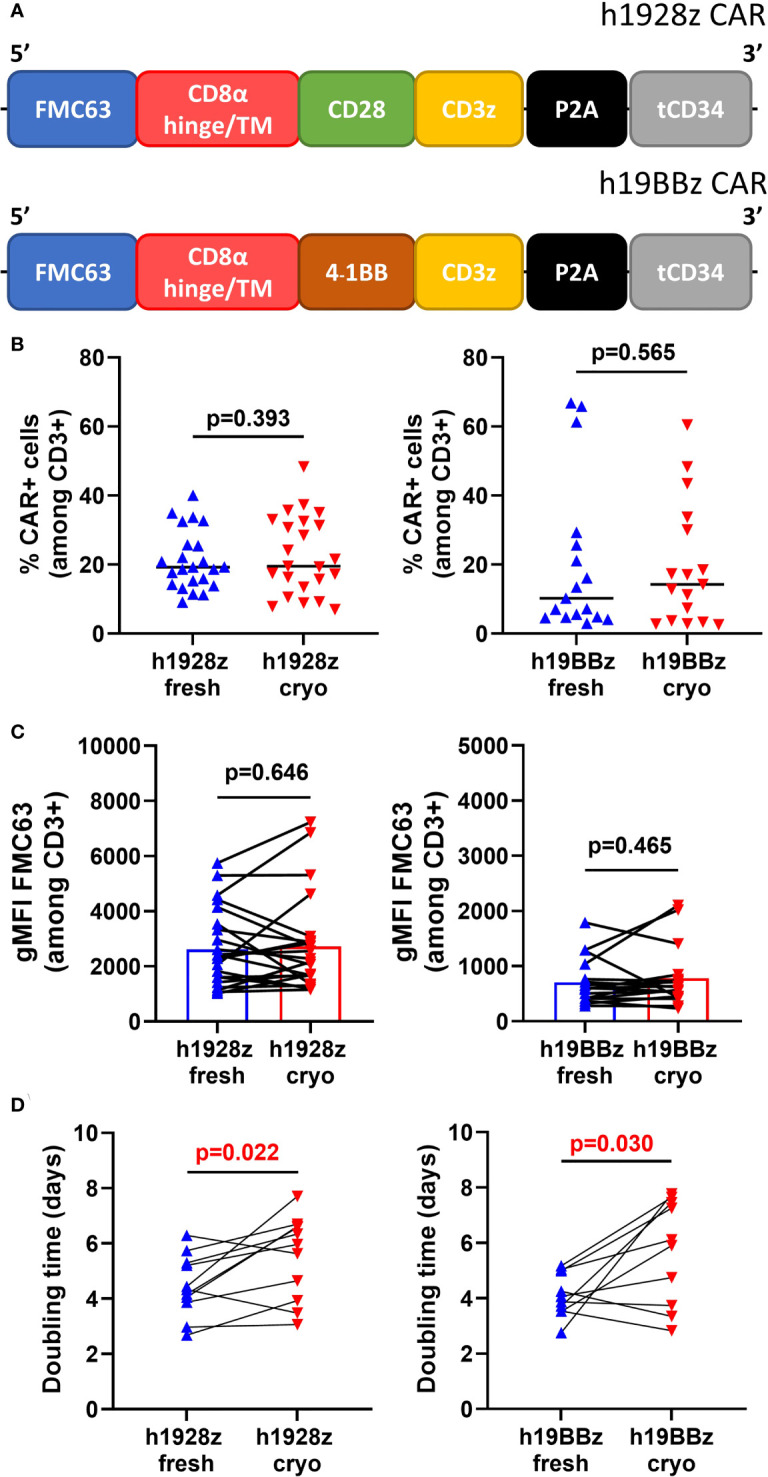
Freezing slows CAR-T cells expansion. **(A)** Schematic representation of h1928z and h19BBz CAR constructs. **(B–D)** Fresh and frozen PBMCs collected from healthy donor buffy coats were used to manufacture h1928z and h19BBz CAR-T cells. 24h activated-PBMCs were transduced with h1928z or h19BBz constructs. After 8-12 days, CAR-T cells manufactured from fresh and frozen PBMCs were collected and frozen (fresh and cryo, respectively). Transduction efficiency **(B)**, CAR density **(C)** and doubling time **(D)** were compared in CAR-T cells rested for 24h before the analyses. Transduction efficiency and CAR density were determined by flow cytometry using Anti-FMC63 mAb (n = independent experiments on 23 (h1928z) and 17 (h19BBz) healthy donors). Doubling time was calculated as day 7/day 1 ratio (n = independent experiments on 11 (h1928z) and 10 (h19BBz) healthy donors). A paired t test was used. Each symbol represents an individual healthy donor, the middle line denoted the median **(A, B)** and the p values are indicated in each graph. A P value ≤ 0.05 was considered significant.

Prior to CAR-T cell manufacture, a patient undergoes leukapheresis to isolate PBMCs that are shipped to the manufacturing facility either fresh or cryopreserved (11). To analyze the effect freezing PBMCs has on the manufacture and function of CAR-T cells, human T cells were genetically modified to express human CD19 directed (hCD19) CARs.). To prepare the CAR-T cells, we used fresh or cryopreserved PBMCs from 30 healthy donors (**Supplementary Figure 1** and **Supplementary Table 1**). The constructs used in this study included two second generation CARs with the same CD19 directed scFv, CD8α hinge and transmembrane domain. One construct contained the cytoplasmic signaling domains (CSD) of CD28 fused to CD3z (h1928z CAR) while the other contained 4-1BB and CD3z CSDs (h19BBz CAR) (**Figure 1A**). After CAR-T manufacture using bulk PBMCs, we first evaluated the impact of cryopreservation on transduction efficiency and CAR density by flow cytometry (**Supplementary Figure 2**). No significant difference was observed in transduction efficiency (**Figure 1B**) and CAR density (**Figure 1C**) between fresh or cryopreserved PBMCs.

Expansion of CAR-T cells *in vitro* is an essential step during the production process, and a longer doubling time during manufacture is associated with worse *in vivo* expansion and efficacy (16). We found that starting production from cryopreserved PBMCs significantly increases the doubling time of CAR-T cells during expansion (**Figure 1D**). The doubling time was 1.25-fold higher in h1928z^cryo^ compared to h1928z^fresh^ CAR-T cells, and 1.5-fold higher in h19BBz^cryo^ versus h19BBz^fresh^ CAR-T cells.

### CAR-T cell product phenotype is not impacted by fresh or frozen starting material

**Figure 2:**
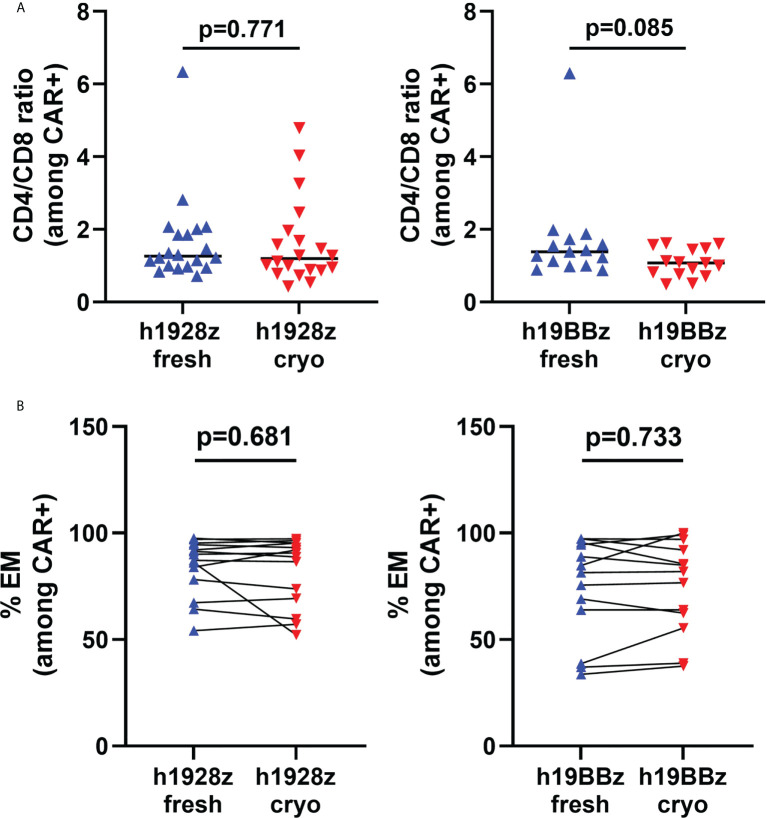
Freezing does not affect phenotype of CAR-T cells. T cells phenotype in CAR-T cells prepared from fresh and cryopreserved PBMC was determined by flow cytometry. **(A)** CD4/CD8 ratio on live CD3+CAR+ cells (n = independent experiments on 20 (h1928z) and 15 (h19BBz) healthy donors). **(B)** Percentage of effector memory (CCR7-CD45RO+) cells in h1928z and h19BBz (n = independent experiments on 14 (h1928z) and 13 (h19BBz) healthy donors). A paired t test was used. Each symbol represents an individual healthy donor, the middle line denoted the median **(A)** and the p values are indicated in each graph. A P value ≤ 0.05 was considered significant.

It has been demonstrated that expansion, persistence, and anti-tumor activity of CAR-T cells is dependent on cell phenotype, with a less differentiated phenotype being associated with superior function in adoptive cell therapy (17, 18). First, we analyzed the CD4/CD8 ratio among CD3+CAR+ cells (**Figure 2A**) and did not detect differences in the ratio between CAR-T cells manufactured from fresh and cryopreserved PBMC. We next analyzed the differentiation state after production, by flow cytometry staining for CCR7 and CD45RO. We explored the percentages of stem central memory (CCR7+CD45RO-), central memory +), effector memory (CCR7-CD45RO+) and terminally differentiated effector cells (CCR7-CD45RO-). We found that starting from cryopreserved PBMCs did not affect cell phenotype in either h1928z or h19BBz CAR-T cells (**Figure 2B**, and **Supplementary Figures 3, 4**). This suggests that the process of cryopreservation of PBMCs does not intrinsically impact the differentiation state of T cells or their ability to differentiate upon activation after thawing.

To validate these results using a different manufacturing process, we prepared h1928z CAR-T cells starting from T cells enriched using magnetic beads. We confirmed our results by showing that there were no differences in CAR transduction efficiency, CD4/CD8 ratio and T cell phenotype when comparing CAR-T cells prepared from fresh versus cryopreserved PBMC from six healthy donors, with a consistently prolonged CAR-T doubling time when starting manufacture from frozen T cells (**Supplementary Figure 5**).

### Cryopreservation of CAR-T cell manufacture starting material does not impact susceptibility to activation induced cell death or mitochondrial dynamics

**Figure 3:**
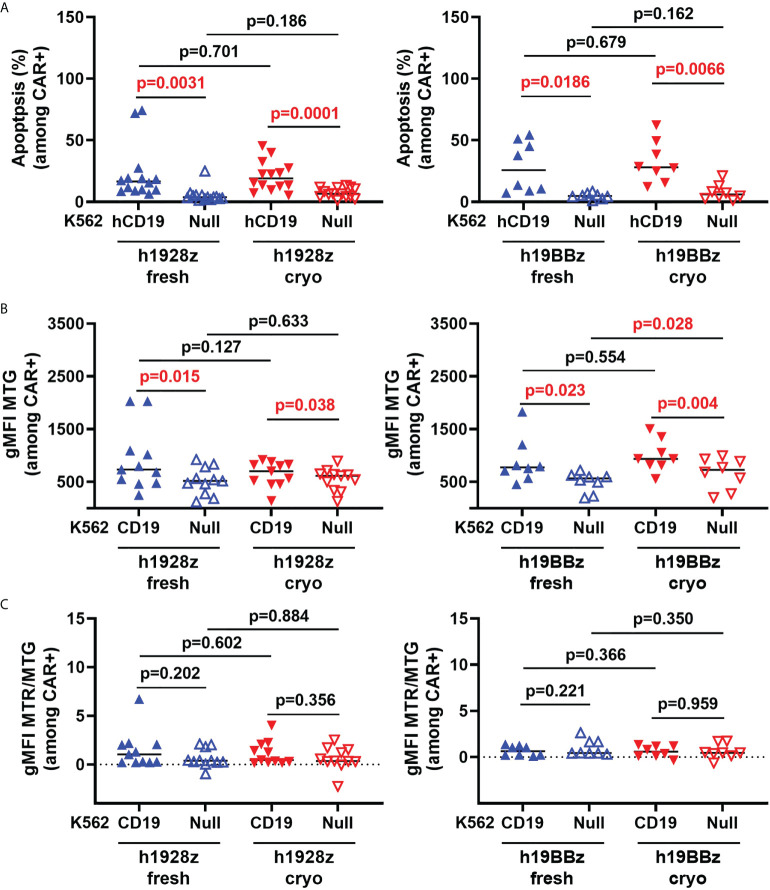
CAR-T cell activation induced cell death and mitochondrial biomass is not altered by freezing. CAR-T cells were stimulated with irradiated hCD19 target cells for 72h. Cells were then collected, and activation induced cell death: AICD **(A)**, mitochondrial biomass **(B)** and mitochondrial quality **(C)** of h1928z (left) and h19BBz (right) were analyzed by flow cytometry. **(A)** n = 2 independent experiments on 14 (h1928z) and eight (h19BBz) healthy donors. **(B)** n = 2 independent experiments on 11 (h1928z) and eight (h19BBz) healthy donors). **(C)** n = 2 independent experiments on 11 (h1928z) and eight (h19BBz) healthy donors. A paired t test was used. Each symbol represents an individual healthy donor, the middle line denoted the median and the p values are indicated in each graph. A P value ≤ 0.05 was considered significant.

Repeated stimulation of CAR-T cells can trigger activation induced cell death (AICD) characterized by the upregulation of death receptors and FasL which mediate extrinsic apoptosis pathways reducing CAR-T cell persistence (19). To determine if cryopreservation sensitizes CAR-T cells to AICD, the percentage of apoptotic cells was quantified by flow cytometry after stimulation with K562 cells expressing human CD19 (hCD19) (**Supplementary Figure 6A**). As expected, CAR-T cells experienced AICD, however no difference in the percentage of apoptotic cells was observed between CAR-T cells derived from fresh and cryopreserved PBMC (**Figure 3A**).

Mitochondrial homeostasis is critical for CAR-T cell survival and function (20). Induction of mitochondrial biogenesis and mitophagy are critical for maintaining mitochondrial health to meet ATP demand of activated CAR -T cells. Using a combination of MitoTracker dyes (**Supplementary Figure 6B**), we analyzed if the mitochondrial dynamics of CAR-T cells was affected by starting manufacture with fresh versus cryopreserved PBMC. MitoTracker red (MTR) stains mitochondria in live cells and its accumulation is dependent upon membrane potential while MitoTracker Green (MTG) is membrane potential-independent and detects changes in mitochondrial biomass. We compared mitochondrial biomass in CAR-T cells (fresh versus cryo) upon activation with CD19-targeted cells. No difference in the MTG intensity was observed (**Figure 3B**) suggesting mitochondrial biogenesis in CAR-T cells is not affected upon antigen engagement by starting manufacture with cryopreserved material. The ratio of MTR to MTG provides a measure of mitochondrial quality by normalizing membrane potential dependent staining, a proxy for functioning mitochondria, to total mitochondrial biomass. We showed that cryopreserving PBMCs before manufacture does not affect the mitochondrial quality control in h1928z or h19BBz in CD3+CAR+ cells compared to manufacturing CAR-Ts from fresh PBMCs as observed by comparable MTR/MTG ratios (**Figure 3C**).

### Manufacturing CAR-T cells from frozen PBMC starting material results in altered cytokine profile with no impairment to CAR-T cell cytotoxic capabilities

**Figure 4:**
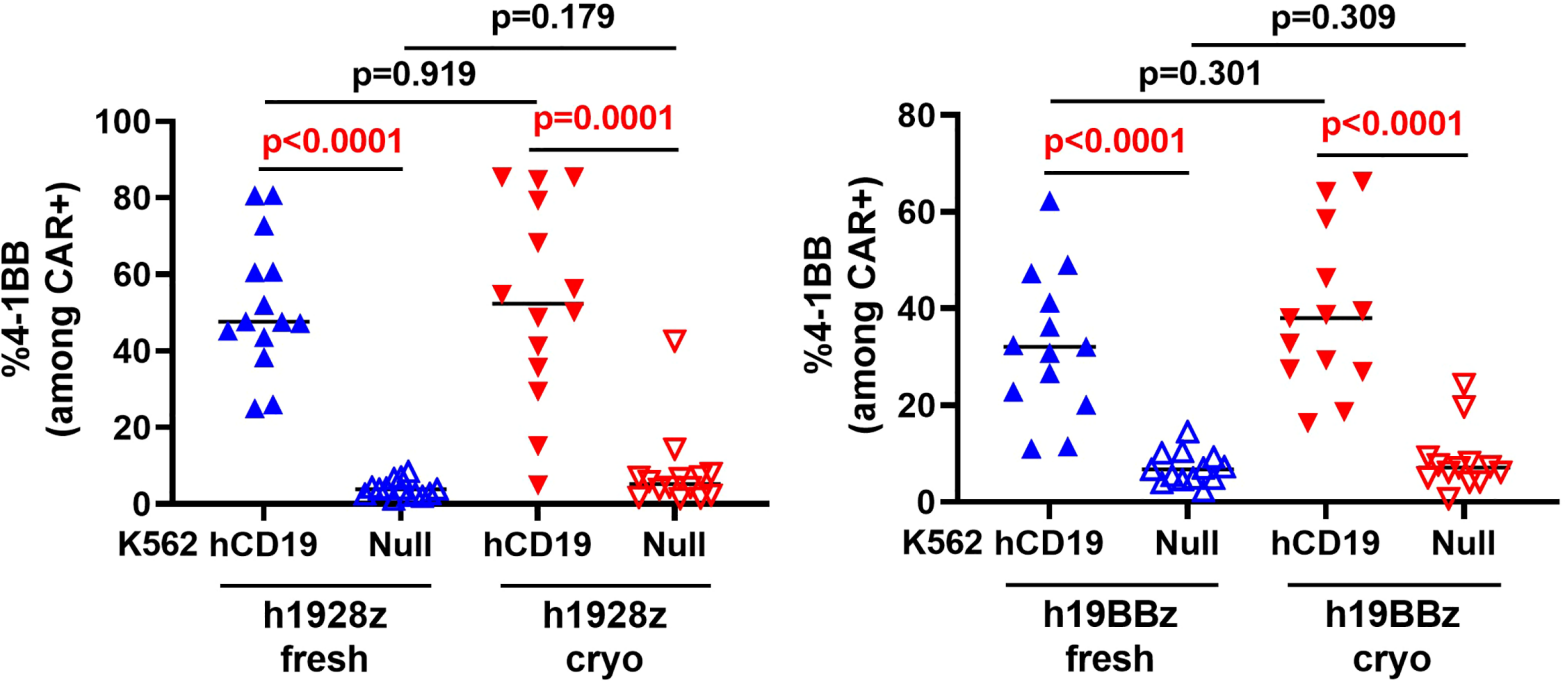
CAR-T cells activation is not altered by freezing. h1928z (left) and h19BBz (right) CAR-T cells were stimulated with hCD19 target cells for 72 h before cells were collected, washed, stained and analyzed by flow cytometry to detect 4-1BB (n = 2 independent experiments on 14 (h1928z) and 13 (h19BBz) healthy donors). A paired t test was used. Each symbol represents an individual healthy donor, the middle line denoted the median and the p values are indicated in each graph. A P value ≤ 0.05 was considered significant.

To explore the effect of cryopreserved PBMCs on CAR-T cell function *in vitro*, we stimulated CAR-T cells with target cells expressing hCD19 and measured expression of the activation marker 4-1BB, cytokine release and cytolytic capacity. 4-1BB expression in T cells is a result of their activation upon stimulation with a cognate antigen MHC complex (21) which also acts as a surrogate for scFv binding and CAR-T cell activation. Here, we compared 4-1BB expression by flow cytometry in stimulated and unstimulated CAR-T cells derived from fresh and cryopreserved PBMC (**Supplementary Figure 6C**). As anticipated, both h1928z and h19BBz CAR-T cells significantly upregulated 4-1BB expression upon activation; however, there were no significant differences between fresh and cryo CAR-T cells (**Figure 4**). This result suggests that cryopreserving PBMCs before CAR-T manufacture does not affect scFv antigen binding, CAR signaling intensity or CAR-T cell activation.

**Figure 5:**
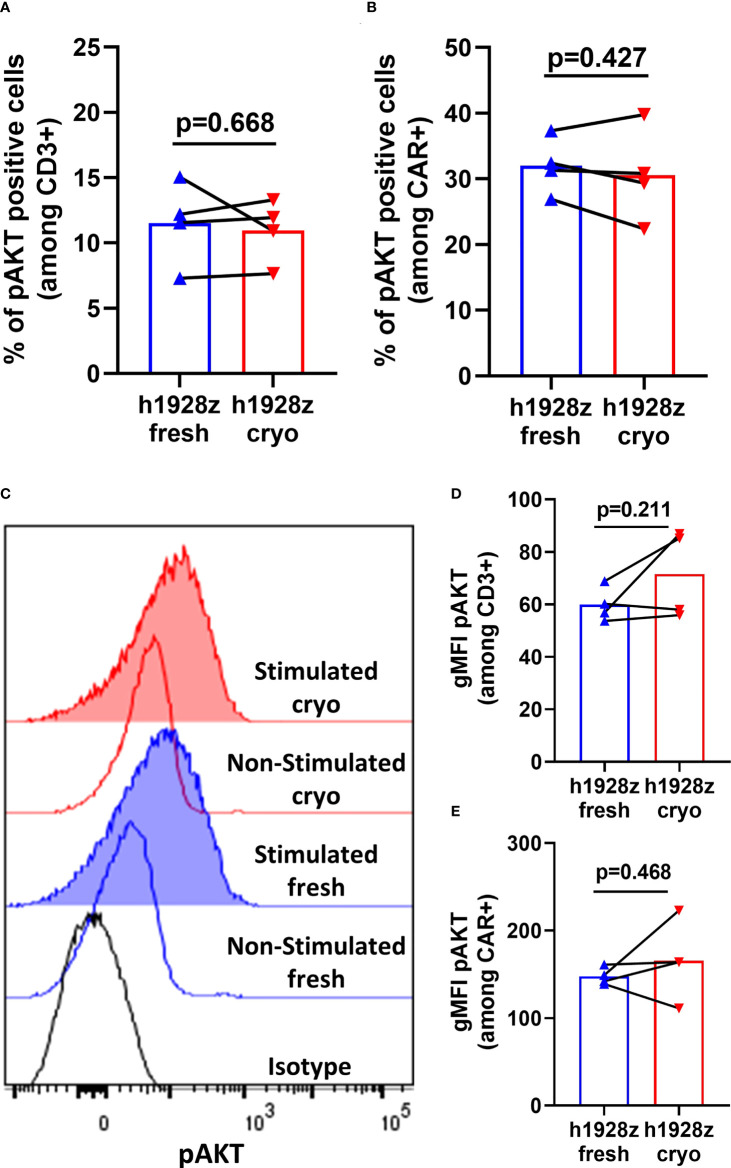
CAR-T cells do not exhibit differences in AKT phosphorylation in fresh and cryo conditions. CAR-T cells were stimulated with OCI-LY3 target cells for 20 minutes and analyzed by phosflow cytometry for AKT phosphorylation. **(A, B)** Percentages of phosphorylated AKT cells within CD3+ and CD3+CAR+ cells (n = 2 independent experiments on four donors). **(C)** Representative histograms for intracellular phospho-AKT staining. Geometric MFIs were obtained for each curve and used to generate the plots presented in **(D, E)**. **(D, E)** Levels of phospho-AKT on CD3+ and CD3+CAR+ cells (n = 2 independent experiments on four healthy donors). A paired t test was used. Each symbol represents an individual healthy donor, and the p values are indicated in each graph. A P value ≤ 0.05 was considered significant.

The AKT pathway is linked to differentiation of T cells from memory to effector phenotype (22, 23) and is key in the development of protective memory CD8+ T cell responses (24). Akt is activated *via* phosphorylation at Serine 308 and Serine 473 downstream of CD3z and CD28 signaling, and its degree of phosphorylation can be used to measure sensitivity of CAR to antigen. Here, we quantified Akt phosphorylation to determine if cryopreservation of PBMCs prior to CAR-T cell manufacture reduced CAR signaling capacity and sensitivity to antigen. h1928z CAR-T cells were stimulated for 20 minutes with OCI-LY3 cells, and un-transduced T cells (UT) were used as a control to determine if phosphorylation was dependent on CAR expression. First, we demonstrated that AKT phosphorylation was not present in untransduced cells to validate pAkt was a result of CAR signaling upon stimulation (**Supplementary Figure 7**). We found CAR-T cell prepared from fresh and cryopreserved PBMC showed comparable percentages and levels of pAKT+ cells when gated both on CD3+ cells and CD3+CAR+ cells (**Figure 5**).

**Figure 6:**
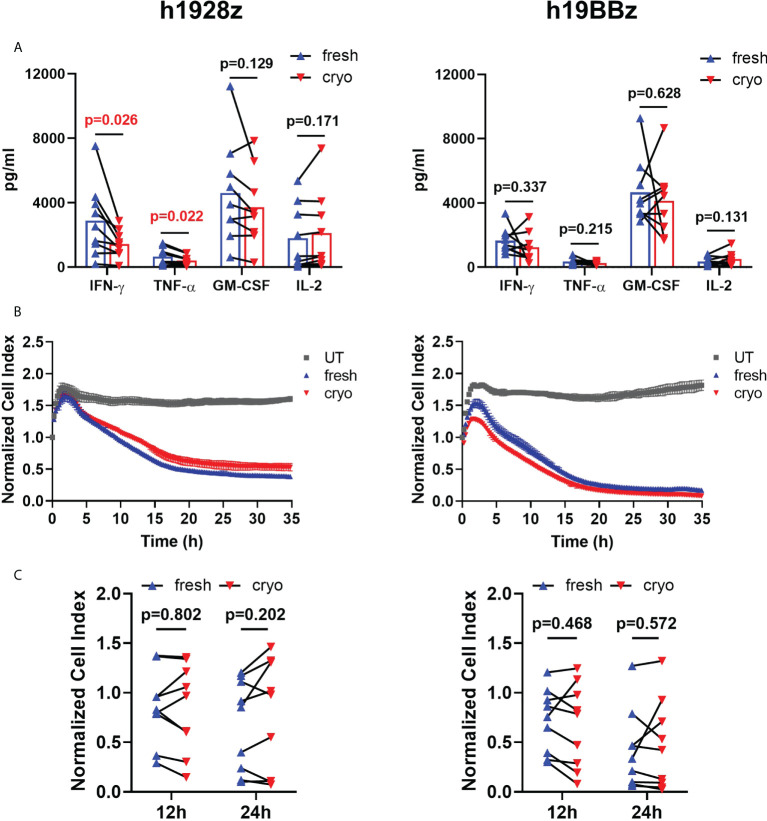
Freezing decreases IFN-γ and TNF-α production on h1928z CAR-T cells but does not affect killing activity. **(A)** CAR-T cells were stimulated with hCD19 target cells for 24 h before supernatant was collected and analyzed by ELLA immunoassay to quantify IFN-γ, TNF-α, GM-CSF and IL-2 (n = 3 independent experiments on nine healthy donors). **(B, C)** The xCELLigence real-time cell analysis system monitored real-time cytotoxicity of CAR-T cells co-cultured with irradiated hCD19 target cells at 1:1 E:T ratio. **(B)** Representative RTCA of h1928z (left) and h19BBz (right) comparing killing activity of untransduced (UT), fresh and cryo CAR-T cells. **(C)** Comparison of the killing activity of CAR-T cells prepared from fresh and cryopreserved PBMC at 12h and 24h (n = 3 independent experiments on nine healthy donors). A paired t test was used. Each symbol represents an individual healthy donor, and the p values are indicated in each graph. A P value ≤ 0.05 was considered significant.

To further compare the effector function of fresh and cryo CAR-T cells, we quantified cytokine secretion after *in vitro* stimulation with hCD19-expressing target cells. We found that there were no differences in IFN-γ, TNF-α, GM-CSF, and IL-2 secretion in h19BBz CAR-T cells when comparing nine healthy donors (**Figure 6A**, right panel). However, h1928z CAR-T cells obtained from cryopreserved PBMCs showed significantly lower levels of IFN-γ and TNF-α compared to fresh CAR-T cells (**Figure 6A**, left panel). These results suggest that co-stimulatory domains drive unique cytokine secretion profiles and in this case 4-1BB may differ in their impact on the ability of CAR-T cells to maintain their capacity to secrete cytokines upon multiple rounds of freezing.

Real time *in vitro* cytolysis experiments showed that fresh and cryo h1928z and h19BBz CAR-T cells lysed target cells with similar kinetics and efficiency (**Figure 6B**). The percentage of target cells lysed at 12h and 24h was not significantly different between CAR-T cells prepared from fresh and cryopreserved PBMC when comparing samples from nine different healthy donors (**Figure 6C**) suggesting cryopreservation did not negatively impact cytolytic capacity. FACS analysis also supported this as degranulation measured by CD107a staining was similar in fresh and cryo samples.

Lastly, we evaluated degranulation by measuring CD107a in h1928z^fresh^ and h1928z^cryo^ CAR-T cells upon 4h-stimulation OCI-LY3 target cells. As expected, here we showed that degranulation is linked to CAR expression (**Supplementary Figure 8A**) and that CAR-T cells had equivalent degranulation levels in fresh and cryo conditions upon stimulation (**Supplementary Figures 8B–D**).

All together these results suggest that *in vitro* anti-tumor function of CAR-T cells is not severely affected by freezing PBMCs prior to manufacture.

## Discussion

CAR-T cell therapies demonstrated remarkable clinical success treating hematological malignancies in several multicenter clinical trials (25, 26). Here, we demonstrate the cryopreservation status of autologous PBMC, the starting material for CAR-T manufacture, impacts *in vitro* IFN-γ, TNF-α secretion (in CD28 second generation CARs) and proliferation during manufacturing but not cytotoxicity. While many features of the manufacturing process differ between FDA approved CAR-T therapies, it is not well established whether fresh versus frozen starting material impacts CAR-T function.

It has been previously described that cryopreserving of PBMC allows for a more flexible CAR-T cell manufacturing process providing greater logistical flexibility to better accommodate scheduling for leukapheresis, shipping, and manufacturing (27, 28). It has also been reported that the frequency and intensity of chemotherapy regimens prior to CAR-T manufacture impair the quality of T cells and ultimately the manufactured CAR-T product, suggesting that T cells cryopreserved from patients prior to extensive chemotherapy or allogeneic donors, attainable only after cryopreservation, could generate a more efficacious product (29). A number of groups have evaluated the effects of cryopreservation with attention to different *in vitro* outcomes of manufacture. Freezing PBMC from patients’ apheresis may deplete suppressive neutrophils (30) and myeloid-derived suppressor cells (31). CAR-T cells with low expression of checkpoint proteins LAG3, TIM-3, PD-1 and TIGIT can be manufactured from frozen peripheral blood even with varying degrees of starting T cell quality (13), suggesting cryopreservation may naturally select fitter T cells from manufacture after thawing (8). Evaluation of CAR-T products across six single-center clinical trials demonstrated there was not a significant difference in expansion, transduction efficiency, or CD4/CD8 ratio at the time of final CAR-T harvest when initiating with fresh or frozen PBMCs (32), with similar *in vivo* expansion, persistence kinetics, and therapeutic efficacy (32).

Here, we manufactured second-generation CAR-T cells from both bulk PBMC and enriched-T cells and found no differences in CAR transduction efficiency, CAR density, CD4/CD8 ratio and immune-phenotype when starting from fresh or cryopreserved material for either h1928z or h19BBz CAR-T cells. In contrast, we found that freezing PBMC increases CAR-T cell doubling time which would likely result in a reduced CAR-T cell dose at the time of infusion. However, we believe the benefits in having more flexibility in scheduling patients’ appointments for leukapheresis and infusion compensates for the increase in time needed for the cells to expand. Future manufacturing processes with rapid and highly efficient methods to reprogram and expand CAR-T cells may circumvent slower expansion associated with cryopreservation, for example rapid manufacture without the need for T cell activation (33).

Once we determined the post manufacture CAR-T cell phenotype was not affected by freezing process, we investigated the impact of cryopreservation on CAR signaling during ligand binding. A recent review summarized the information to date on AICD and CAR-T cell therapy. The upregulation of death receptors that mediate AICD is directly related to several factors including sustained tumor antigen stimulation, among others (19). However, to our knowledge this is the first study showing that starting the manufacturing process from cryopreserved PBMCs did not increase CAR-T cell death upon antigen stimulation compared to cells produced from fresh PBMCs. We also found that mitochondrial biomass and membrane potential, a measure of metabolic fitness, was not affected by the starting material. This is important as the fitness of CAR-T cells could impact function (34) and the therapeutic efficacy (13).

Our data suggest that CAR-T cells, regardless of cryopreservation status before manufacture, should have similar antigen sensitivity and signaling intensities, degranulation, and anti-tumor function. However, secretion of pro-inflammatory cytokines IFN-γ and TNF-α was significantly decreased in CAR-T cells manufactured from cryopreserved PBMC expressing h1928z CARs. These observations corroborate results from a recent study where cryopreservation of starting material for eventual BCMA-CAR-T cells with a CD28 signaling domain showed decreased IL-2, TNF-α and IFN-γ secretion without affecting anti-tumor function (35). Interestingly, this trend was not present in h19BBz CARs. As expected, CD28-based CAR-T cells secreted more IFN-γ, TNF-α and IL-2 compared to 4-1BB-based CAR-T cells upon activation. This is a result stemming from the unique signaling pathways actuated by of CD28 costimulatory domain eliciting stronger T cell activation and cytokine secretion, compared to 4-1BB (36). A longer doubling time during manufacture has been associated with worse *in vivo* expansion and efficacy, while conversely lower IFN-γ secretion associated with better efficacy, were both previously demonstrated in CD19-directed CAR-T cell products manufactured in the ZUMA-1 trial for lymphoma patients which utilized freshly collected apheresis material for manufacture (16). The results of the present study must be taken in context: the impact of freezing the starting material on efficacy and safety may not be captured here, and additional correlative analysis in conjunction with CAR-T treated patients with annotated clinical results is warranted.

There are several limitations of our study. First, we did not investigate PBMC cells cryopreserved for a long period of time since we were doing side-by-side comparison of CAR-T cells from the same individuals. Second, we only included PBMCs from healthy donors, without interrogation of the impact of cryopreservation on PBMCs from patients. We recognize that CAR-T cells manufactured from healthy donors and cancer patients could have different results. However, results from Panch et al. discussed above, suggest that the starting material does not affect many of the commonly interrogated characteristics of CAR-T cell products or function in patients. Other cytogenetic analyses may reveal distinct impacts of cryopreservation on epigenetic landscapes in cryopreserved versus freshly manufactured CAR-T cells. Third, there are endless possible iterations of manufacturing conditions which could not all be tested. For example, we did not compare conditions of frozen PBMC immediately activated for viral transduction versus those having been thawed and rested in culture media for 24 hours, although cell numbers were normalized after activation. Fourth, we did not perform a complete robust analysis of metabolic function in CAR-T cells manufactured from fresh and cryopreserved PBMC. Metabolic fitness may have a significant impact on proliferative capacity and persistence. Finally, we did not perform *in vivo* experiments. However, our *in vitro* results correlate with those showed by Panch et al. suggesting that cryopreservation the starting material and the final CAR-T product does not affect the final product.

## Conclusion

Cryopreservation of PBMCs prior to CAR-T cells production slows cell expansion during manufacture, but does not affect CAR-T cell phenotype, activation, or *in vitro* anti-tumor function of h1928z or h19BBz. As CAR-T immunotherapy continues to expand globally, the need to store PBMCs to improve manufacturing logistics and capacity, as well as to allow collection from patients at an early clinical stage will grow. This study helps support the concept that cryopreservation of PBMCs is a valid solution to these issues without compromising the quality of the final cellular product.

## Data availability statement

The original contributions presented in the study are included in the article/**Supplementary Material**. Further inquiries can be directed to the corresponding author.

## Author contributions

FLL conceived of the project. JAM and FLL were involved in the experimental design and writing the first and final manuscript drafts. JAM, MMe, RA, MMa, JK, JT, MHV and FLL were involved in assay design and optimization. JAM and MMe performed the experiments and performed initial data analysis with FLL. All authors reviewed data and contributed to the article review and editing and approved the submitted version.

## Funding

This work was funded in part by Kite Pharma and a donation from the Hyer family to the Moffitt Foundation. FL is supported as a Leukemia and Lymphoma Society Clinical Scholar. This work was supported by the flow cytometry core facility of Moffitt Cancer Center (P30-CA076292).

## Acknowledgments

The authors thank Adrian Bot and John Rossi from Kite Pharma for helpful and critical scientific discussions, and the Flow Cytometry Core at Moffitt Cancer Center.

## Conflict of interest

MMa and JK are employed by Kite Pharma, one of the funders of this work. Kite Pharma was involved in the study design, analysis, and interpretation of data. FLL served as a scientific advisor to: A2, Allogene, Amgen, Bluebird Bio, BMS/Celgene, Calibr, Caribou, Cellular Biomedicine Group, Daiichi Sankyo, GammaDelta Therapeutics, Iovance, Kite Pharma, Janssen, Legend Biotech, Novartis, Sana, Takeda, Wugen, Umoja; received research funding from: Kite Pharma (Institutional), Allogene (Institutional), CERo Therapeutics (Institutional), Novartis (Institutional), BlueBird Bio (Institutional), BMS (Institutional), National Cancer Institute, Leukemia and Lymphoma Society; has patents held by the institution in his name (unlicensed) in the field of cellular immunotherapy; has served as a consultant to: Cowen, EcoR1, Emerging Therapy Solutions, Gerson Lehrman Group (GLG); and has been compensated for educational activities by: Aptitude Health, ASH, BioPharma Communications CARE Education, Clinical Care Options Oncology, Imedex, Society for Immunotherapy of Cancer.

The remaining authors declare that the research was conducted in the absence of any commercial or financial relationships that could be construed as a potential conflict of interest.

## Publisher’s note

All claims expressed in this article are solely those of the authors and do not necessarily represent those of their affiliated organizations, or those of the publisher, the editors and the reviewers. Any product that may be evaluated in this article, or claim that may be made by its manufacturer, is not guaranteed or endorsed by the publisher.
