# Supplementary material for: CAR-T manufactured from frozen PBMC yield efficient function with prolonged in vitro production
Source: Front Immunol. 2022 Sep 26;13:1007042. doi: 10.3389/fimmu.2022.1007042 (PMC9549966; doi:10.3389/fimmu.2022.1007042)
Supplement: Supplementary file 1 [file DataSheet_1.docx]

Supplementary Material


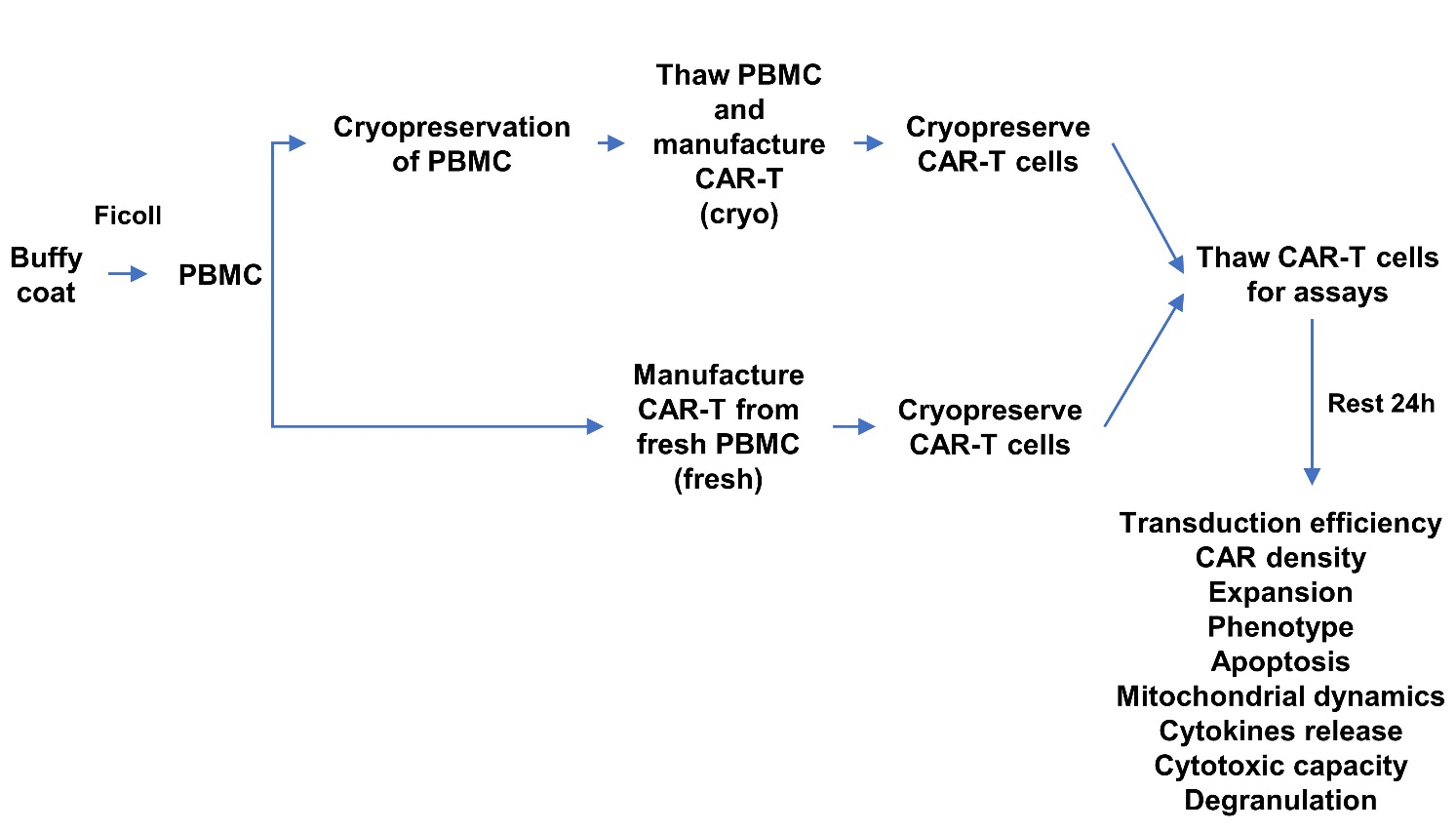


**Supplementary Figure 1.** **CAR-T cell manufacture schematic representation.** PBMCs were collected from healthy donor buffy coats after ficoll process. Cells were either used fresh to prepare CAR-T cells or cryopreserved to prepare CAR-T cells from cryopreserved PBMC. After CAR-T cells expansion, they were cryopreserved, and then thawed 24h prior experimental analysis.

**
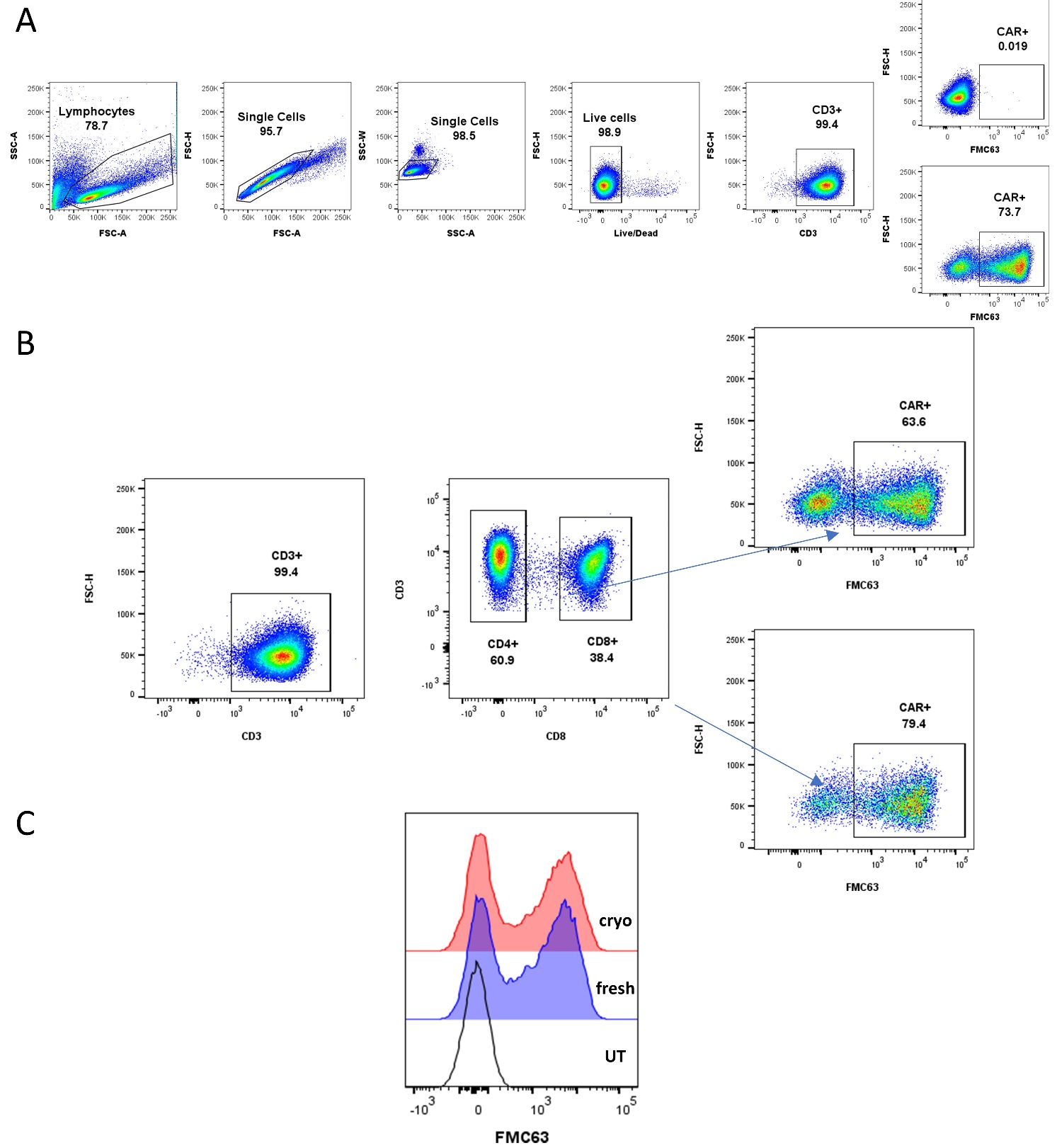
**

**Supplementary Figure 2. Flow gating strategy.** CAR-T cells transduction efficiency was determined among CD3+ (A), CD4+, and CD8+ (B) cells using an anti-FMC63 scFv antibody designed by Acrobiosystems. Untransduced (UT) cells were used to determine the CAR negative cells. (C) Representative histograms for FMC63 staining. Geometric MFIs were obtained for each curve and used to generate the plots presented in Figure 1C.

**
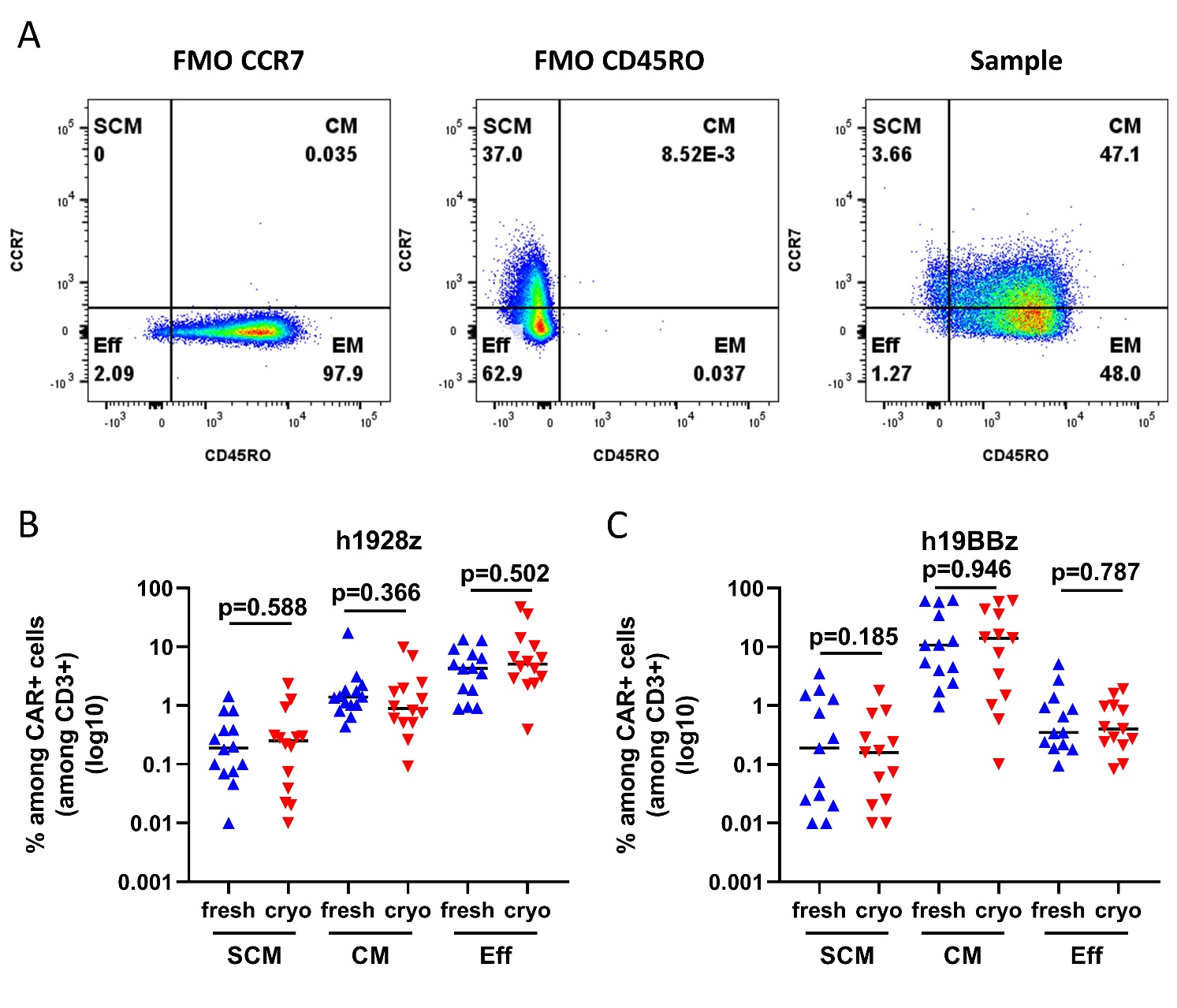
**

**Supplementary Figure 3. Gating strategy and phenotype of CAR-T cells.** (A) CAR-T cells phenotype was analyzed by flow cytometry using anti-CCR7 and anti-CD45RO antibodies to determine stem central memory (CCR7+CD45RO-), central memory (CCR7+CD45RO+), and terminally differentiated effector (CCR7-CD45RO-) cells. (B-C) CAR-T cells phenotype in h1928z (B) and h19BBz (C). Effector memory (CCR7-CD45RO+) cells comparison is displayed in Figure 2B. n = independent experiments on 14 (h1928z) and 13 (h19BBz) healthy donors. A paired t test was used. Each symbol represents an individual healthy donor, the middle line denoted the median and the p values are indicated in each graph. A P value ≤ 0.05 was considered significant.

**
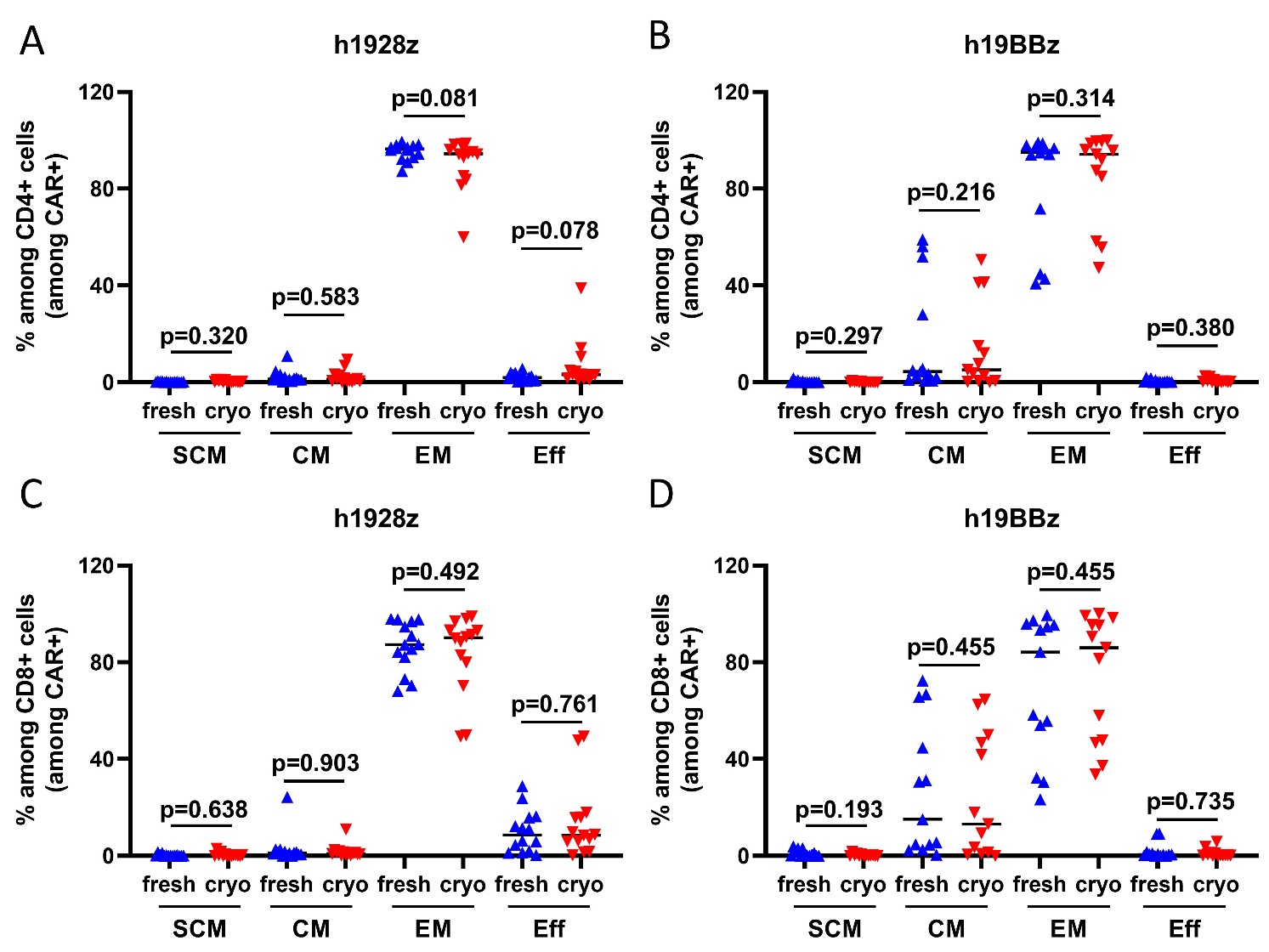
**

**Supplementary Figure 4. Phenotype of CD4+ and CD8+ CAR-T cells.** CD4+ and CD8+ phenotype in CAR-T cells was determined by flow cytometry. (A-D) Stem Central Memory (CCR7+CD45RO-), central memory (CCR7+CD45RO+), effector memory (CCR7-CD45RO+) and terminally differentiated effector (CCR7-CD45RO-) phenotype in h1928z (A, C) and h19BBz (B, C). n = independent experiments on 14 (h1928z) and 13 (h19BBz) healthy donors. A paired t test was used. Each symbol represents an individual healthy donor, the middle line denoted the median and the p values are indicated in each graph. A P value ≤ 0.05 was considered significant.

**
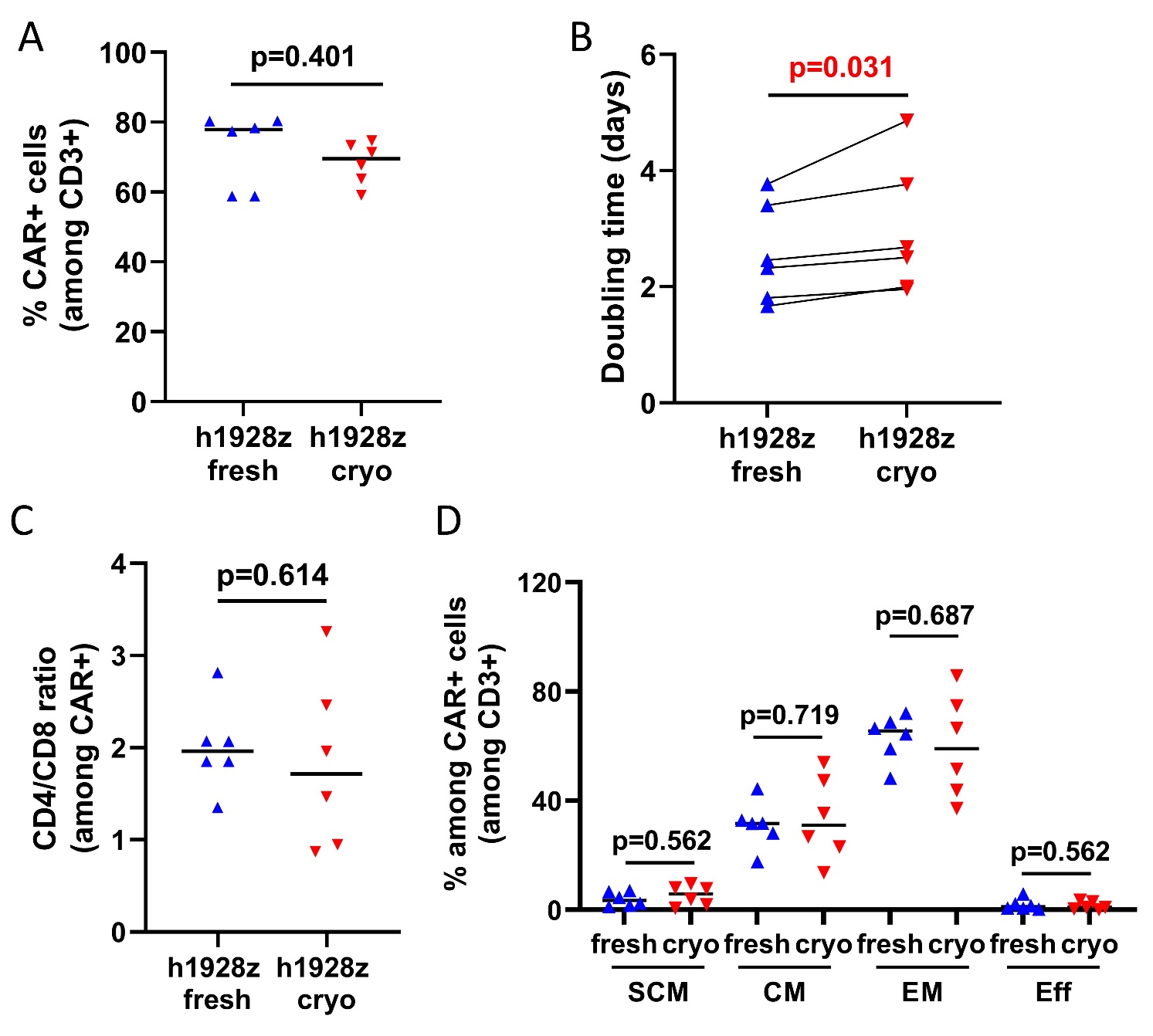
**

**Supplementary Figure 5. Comparison between CAR-Ts manufactured from fresh and cryopreserved PBMCs.** (A-D) Fresh and cryopreserved PBMCs collected from healthy donor buffy coats were used to enrich T cells. 24h activated-T cells were transduced with h1928z. After 8-12 days, CAR-T cells manufactured from fresh and cryopreserved PBMCs were collected and frozen (fresh and cryo, respectively). CAR expression (A), doubling time (B), and T cells phenotype (C-D) in fresh and cryo CAR-T cells were compared in cells rested for 24h before the analyses. (D) Stem Central Memory (CCR7+CD45RO-), central memory (CCR7+CD45RO+), effector memory (CCR7-CD45RO+) and terminally differentiated effector (CCR7-CD45RO-) phenotype. n = 3 independent experiments on 6 healthy donors. A paired t test was used. Each symbol represents an individual healthy donor, the middle line denoted the median and the p values are indicated in each graph. A P value ≤ 0.05 was considered significant.


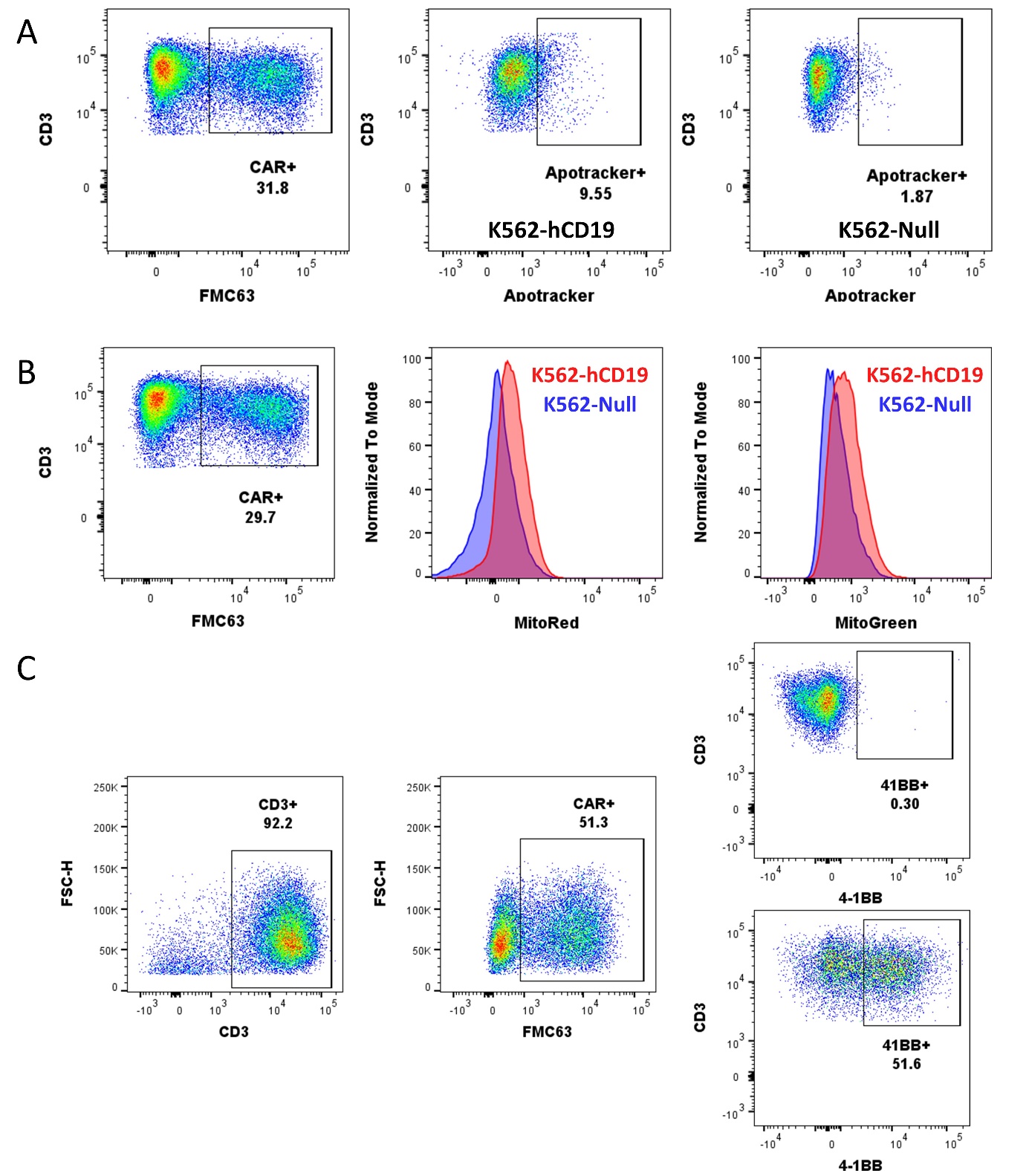


**Supplementary Figure 6. Flow gating strategy.** (A) Activation induced cell death (AICD) was determined among CD3+CAR+ cells using Apotracker™ Green (BioLegend). (B) MitoTracker Red and Green (Invitrogen™) were used to determine the metabolic fitness of the cells by analyzing the membrane potential (red) and mass (green) by flow cytometry. (C) 4-1BB expression among CD3+CAR+ cells was determined by flow cytometry using an anti-4-1BB antibody.

**
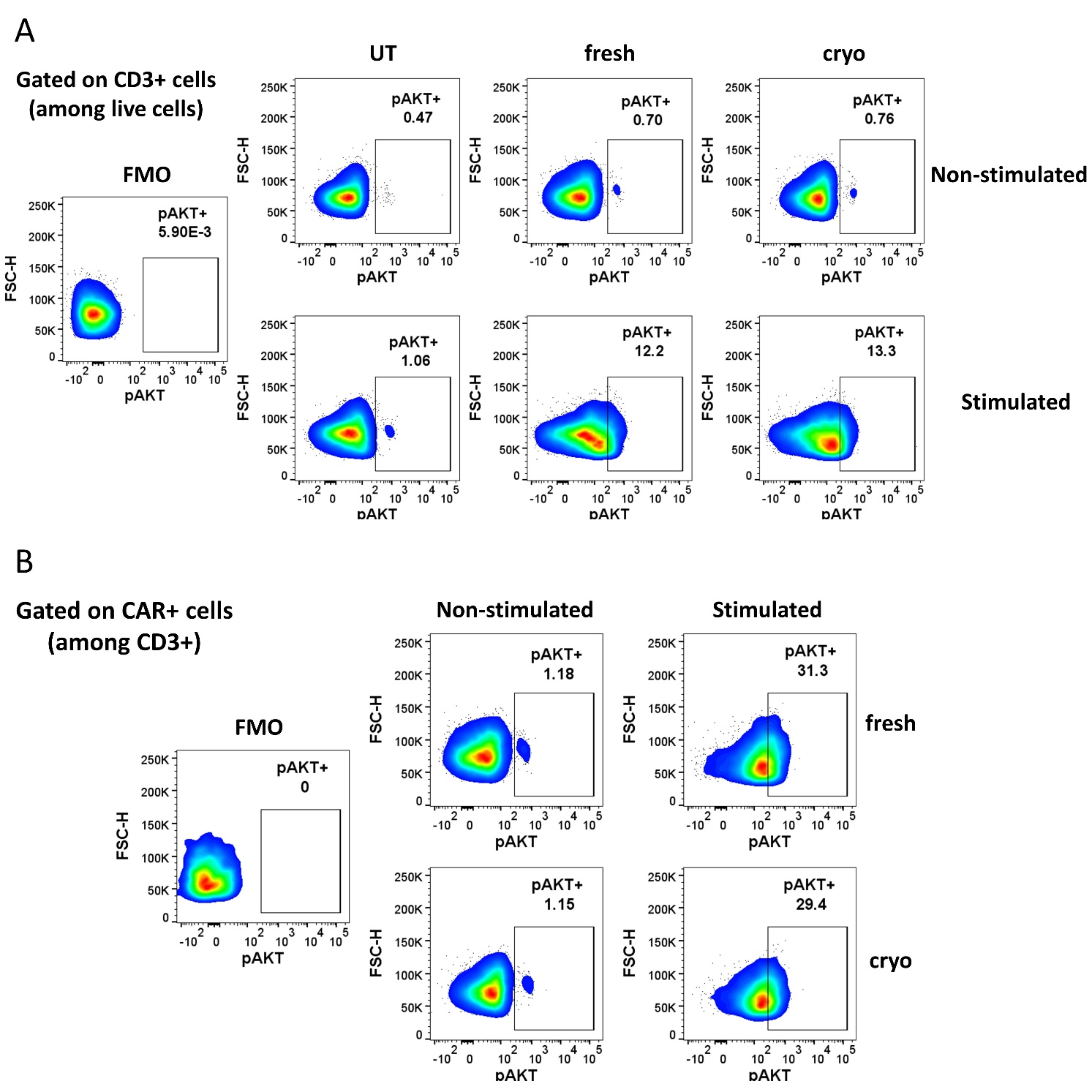
**

**Supplementary Figure 7. AKT phosphorylation is not affected by freezing PBMCs.** CAR-T cells were stimulated with OCI-LY3 target cells for 20 minutes and analyzed by phosflow cytometry for AKT phosphorylation. Representative flow cytometry analysis showing intracellular phospho-AKT positive cells within CD3+ (A) and CD3+CAR+ (B) cells. FMO: fluorescence minus one control.

**
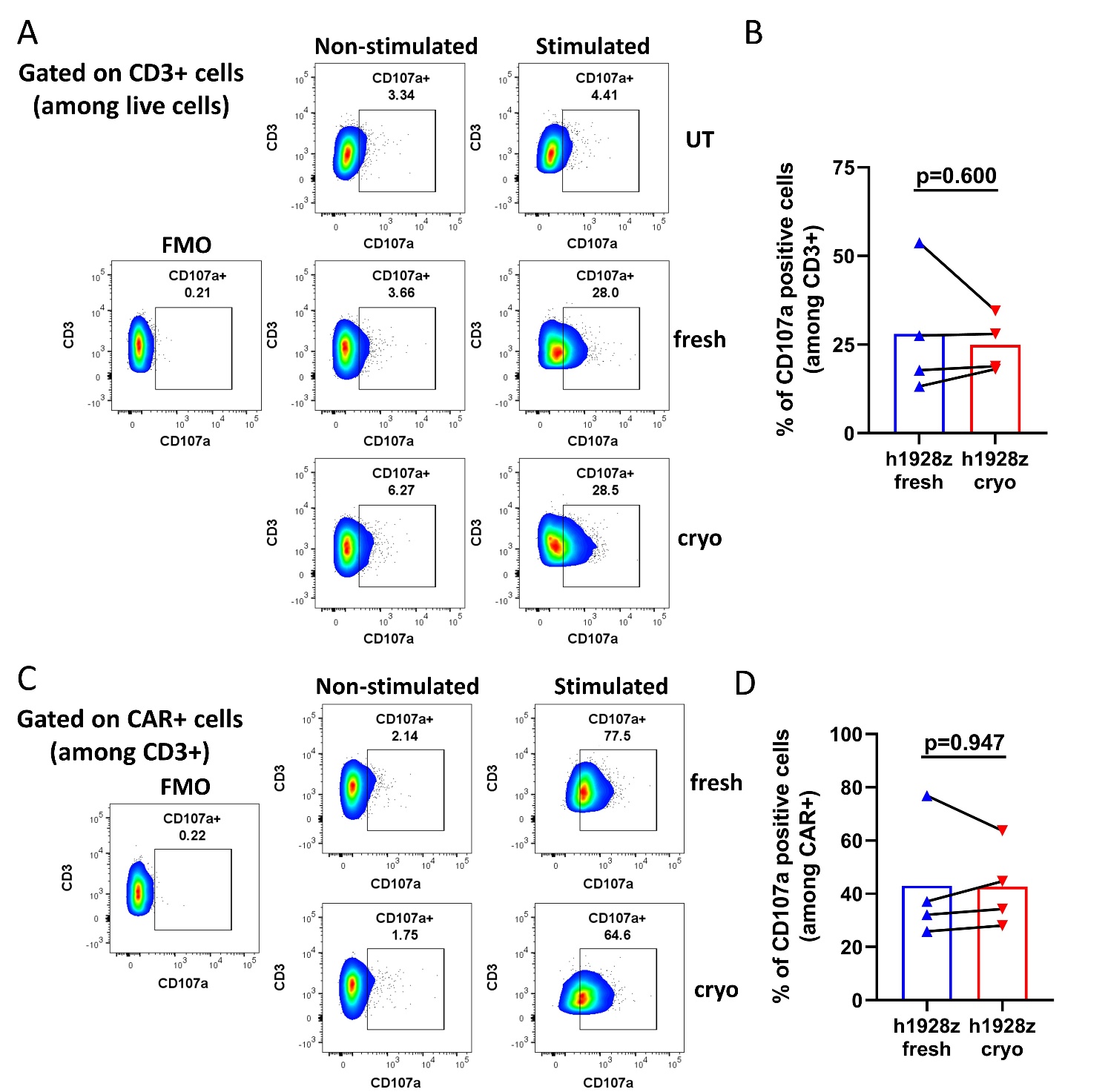
**

**Supplementary Figure 8. CAR-T cells manufactured from fresh and cryopreserved PBMC exhibit similar degranulation upon stimulation.** Degranulation in was measured by CD107a staining upon incubation of h1928z CAR-T cells with OCI-LY3 target cells for 4h. Representative flow cytometry analysis showing CD107a positive cells within CD3+ (A) and CD3+CAR+ (C) cells. FMO: fluorescence minus one control. Percentages of CD107a cells within CD3+ (B) and CD3+CAR+ (D) cells. A paired t test was used. Each symbol represents an individual healthy donor (n = 2 independent experiments on 4 healthy donors), and the p values are indicated in each graph. A P value ≤ 0.05 was considered significant.

**Supplementary Table 1. Healthy donor characteristics**

| **HD** | **Age** | **Gender** |
| --- | --- | --- |
| 1 | 30 | Male |
| 2 | 45 | Male |
| 3 | 69 | Male |
| 4 | 66 | Male |
| 5 | 22 | Female |
| 6 | 72 | Male |
| 7 | 30 | Female |
| 8 | 52 | Female |
| 9 | 66 | Male |
| 10 | 80 | Female |
| 11 | 71 | Male |
| 12 | 68 | Female |
| 13 | 74 | Male |
| 14 | 18 | Male |
| 15 | 43 | Male |
| 16 | 28 | Male |
| 17 | 22 | Female |
| 18 | 20 | Female |
| 19 | 20 | Female |
| 20 | 41 | Female |
| 21 | 57 | Male |
| 22 | 41 | Male |
| 23 | 63 | Male |
| 24 | 32 | Female |
| 25 | 63 | Male |
| 26 | 49 | Male |
| 27 | 32 | Male |
| 28 | 42 | Male |
| 29 | 56 | Female |
| 30 | 35 | Male |

**Supplementary Table 2. h1928z CAR-T cells**

| **Fig**  **HD** | **1B** | **1C** | **1D** | **2A** | **2B** | **3A** | **3B** | **3C** | **4** | **5** | **6** | **Sup 3** | **Sup 4** | **Sup 5** | **Sup 8** |
| --- | --- | --- | --- | --- | --- | --- | --- | --- | --- | --- | --- | --- | --- | --- | --- |
| 1 | X | X |  |  |  | X | X | X | X |  |  |  |  |  |  |
| 2 | X | X |  | X |  | X | X | X | X |  |  |  |  |  |  |
| 3 | X | X |  | X |  | X | X | X | X |  |  |  |  |  |  |
| 4 | X | X |  | X |  | X | X | X | X |  |  |  |  |  |  |
| 5 | X | X |  |  |  | X |  |  | X |  |  |  |  |  |  |
| 6 | X | X |  |  |  | X |  |  | X |  |  |  |  |  |  |
| 7 | X | X | X | X |  | X | X | X | X |  |  |  |  |  |  |
| 8 | X | X | X | X |  | X | X | X | X |  |  |  |  |  |  |
| 9 | X | X | X | X |  | X | X | X | X |  |  |  |  |  |  |
| 10 | X | X | X | X | X | X | X | X | X |  |  | X | X |  |  |
| 11 | X | X | X | X | X | X | X | X | X |  |  | X | X |  |  |
| 12 | X | X | X | X | X |  | X | X |  |  |  | X | X |  |  |
| 13 | X | X | X | X | X | X |  |  | X |  |  | X | X |  |  |
| 14 | X | X | X | X | X | X | X | X | X |  |  | X | X |  |  |
| 15 |  |  | X |  |  | X |  |  | X |  |  |  |  |  |  |
| 16 | X | X | X | X | X |  |  |  |  |  | X | X | X |  |  |
| 17 | X | X | X | X | X |  |  |  |  |  | X | X | X |  |  |
| 18 | X | X |  | X | X |  |  |  |  |  | X | X | X |  |  |
| 19 | X | X |  | X | X |  |  |  |  |  | X | X | X |  |  |
| 20 | X | X |  | X | X |  |  |  |  |  | X | X | X |  |  |
| 21 | X | X |  | X | X |  |  |  |  | X | X | X | X |  | X |
| 22 | X | X |  | X | X |  |  |  |  | X | X | X | X |  | X |
| 23 | X | X |  | X | X |  |  |  |  | X | X | X | X |  | X |
| 24 | X | X |  | X | X |  |  |  |  | X | X | X | X |  | X |
| 25 |  |  |  |  |  |  |  |  |  |  |  |  |  | X |  |
| 26 |  |  |  |  |  |  |  |  |  |  |  |  |  | X |  |
| 27 |  |  |  |  |  |  |  |  |  |  |  |  |  | X |  |
| 28 |  |  |  |  |  |  |  |  |  |  |  |  |  | X |  |
| 29 |  |  |  |  |  |  |  |  |  |  |  |  |  | X |  |
| 30 |  |  |  |  |  |  |  |  |  |  |  |  |  | X |  |

**Supplementary Table 3. h19BBz CAR-T cells**

| **Fig**  **HD** | **1B** | **1C** | **1D** | **2A** | **2B** | **3** | **4** | **6** | **Sup 3** | **Sup 4** |
| --- | --- | --- | --- | --- | --- | --- | --- | --- | --- | --- |
| 1 | X | X |  |  |  |  | X |  |  |  |
| 2 | X | X |  |  |  |  | X |  |  |  |
| 3 | X | X |  |  |  | X |  |  |  |  |
| 4 |  |  |  |  |  | X | X |  |  |  |
| 5 |  |  |  |  |  |  | X |  |  |  |
| 6 |  |  |  |  |  | X | X |  |  |  |
| 7 |  |  | X |  |  | X | X |  |  |  |
| 8 | X | X | X |  |  | X | X |  |  |  |
| 9 | X | X | X | X |  | X | X |  |  |  |
| 10 | X | X | X | X | X |  | X |  | X | X |
| 11 | X | X | X | X | X | X | X |  | X | X |
| 12 |  |  | X | X |  | X |  |  |  |  |
| 13 |  |  | X | X | X |  | X |  | X | X |
| 14 |  |  |  | X | X |  | X |  | X | X |
| 15 | X | X | X |  |  |  | X |  |  |  |
| 16 | X | X | X | X | X |  |  | X | X | X |
| 17 | X | X | X | X | X |  |  | X | X | X |
| 18 | X | X |  | X | X |  |  | X | X | X |
| 19 | X | X |  | X | X |  |  | X | X | X |
| 20 | X | X |  | X | X |  |  | X | X | X |
| 21 | X | X |  | X | X |  |  | X | X | X |
| 22 | X | X |  | X | X |  |  | X | X | X |
| 23 | X | X |  | X | X |  |  | X | X | X |
| 24 | X | X |  | X | X |  |  | X | X | X |
